# Supplementary material for: Predicting visual function by interpreting a neuronal wiring diagram
Source: Nature. 2024 Oct 2;634(8032):113–23. doi: 10.1038/s41586-024-07953-5 (PMC11446822; doi:10.1038/s41586-024-07953-5)
Supplement: Supplementary file 6 — CRF and ERF predictions for individual TmY4 and TmY9 cells. Analogous to Supplementary Data 3, but for TmY target types. Shown are the top four monosynaptic pathways, the strongest pathway passing through each of the top ten intermediary types (ranking from Extended Data Fig. 7), and the trisynaptic pathway Tm1–TmY–Dm3–TmY (see the section entitled Prediction of spatial normalization). [file 41586_2024_7953_MOESM6_ESM.zip › DataS4/TmY9qΓèÑ/720575940613030422.pdf]

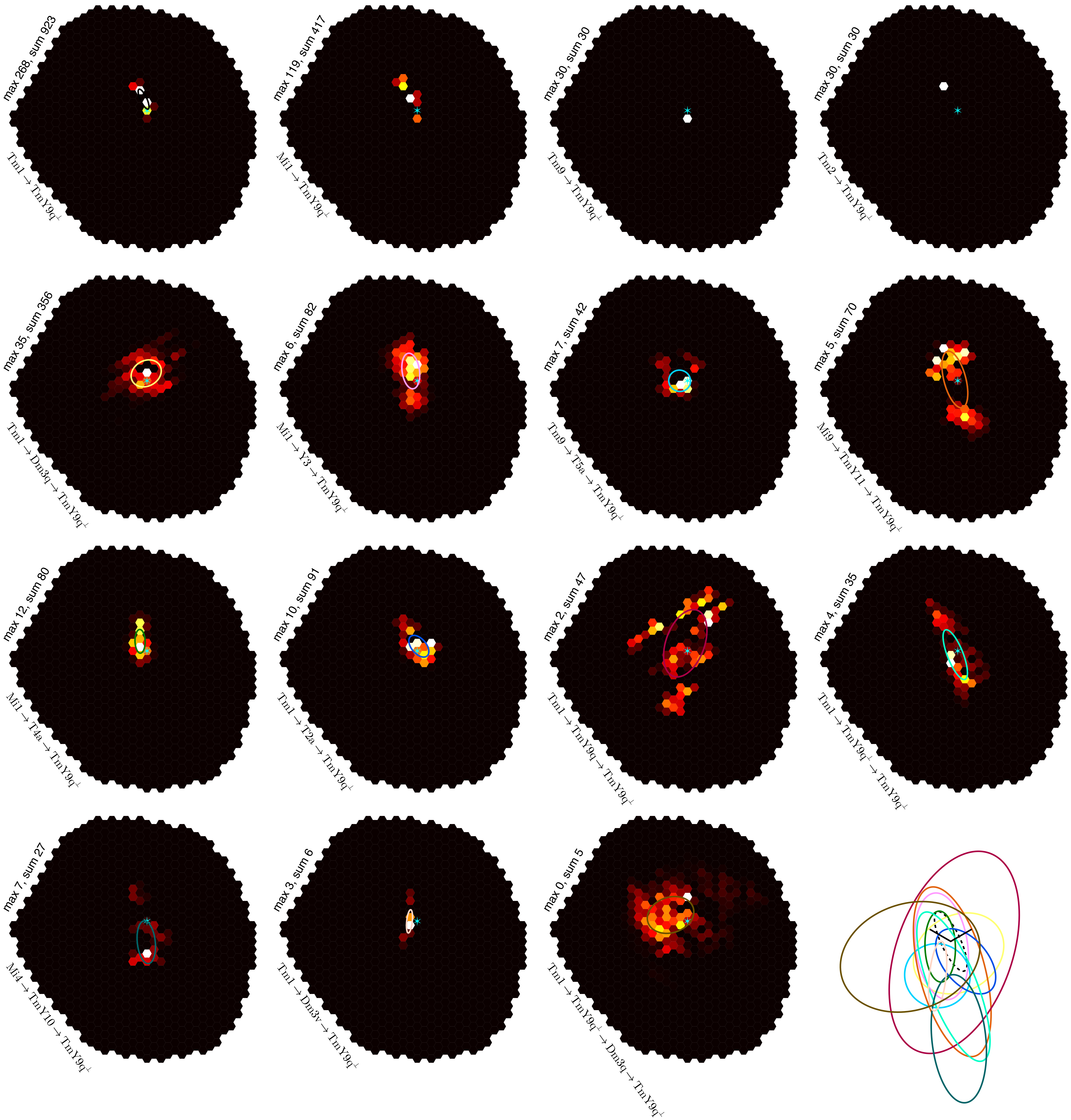
max 268, sum 923
Tm1 → TmY9q⊥
max 119, sum 417
Mi1 → TmY9q⊥
max 30, sum 30
Tm9 → TmY9q⊥
max 30, sum 30
Tm2 → TmY9q⊥
max 35, sum 356
Tm1 → Dm3q → TmY9q⊥
max 6, sum 82
Mi1 → Y3 → TmY9q⊥
max 7, sum 42
Tm9 → T5a → TmY9q⊥
max 5, sum 70
Mi9 → TmY11 → TmY9q⊥
max 12, sum 80
Mi1 → T4a → TmY9q⊥
max 10, sum 91
Tm1 → T2a → TmY9q⊥
max 2, sum 47
Tm1 → TmY9q⊥ → TmY9q⊥
max 4, sum 35
Tm1 → TmY9q⊥ → TmY9q⊥
max 7, sum 27
Mi4 → TmY10 → TmY9q⊥
max 3, sum 6
Tm1 → Dm3v → TmY9q⊥
max 0, sum 5
Tm1 → TmY9q⊥ → Dm3q → TmY9q⊥
